# Measuring and Promoting SARS-CoV-2 Vaccine Equity: Development of a COVID-19 Vaccine Equity Index

**DOI:** 10.1089/heq.2021.0047

**Published:** 2021-07-13

**Authors:** Alice R. Pressman, Stephen H. Lockhart, Zijun Shen, Kristen M.J. Azar

**Affiliations:** ^1^ Sutter Health, Institute for Advancing Health Equity, Walnut Creek, California, USA.; ^2^ Sutter Health, Center for Health Systems Research, Walnut Creek, California, USA.; ^3^ Department of Epidemiology and Biostatistics, University of California, San Francisco, San Francisco, California, USA.

**Keywords:** COVID-19, health equity, vaccine equity, SARS-CoV-2, COVID-19 vaccine equity index

## Abstract

**Purpose:** The coronavirus pandemic has created the greatest public health crisis in a century, causing >500,000 deaths in the United States alone. Minoritized and socioeconomically disadvantaged groups have borne a disproportionate burden of severe illness, hospitalization, and death from COVID-19. Recently developed FDA-approved vaccines have been shown to significantly reduce severe COVID-19–related outcomes. Vaccination campaigns have the potential to advance health equity by prioritizing allocation to those at highest risk while striving for herd immunity. Large integrated health systems have been faced with the daunting task of meeting the rapidly evolving needs of diverse patient populations for the provision of population-based testing, treatment, education, and now vaccine distribution. We have designed a COVID-19 vaccine equity index (CVEI) to guide health system vaccination strategy.

**Methods:** We considered proportion unvaccinated within a health care system. We then used real-time readily available electronic health record (EHR) COVID-19 testing positivity and proportion hospitalized to measure burden of illness by race/ethnicity. We used conditional probability and statistical theory to measure equity for unvaccinated individuals and to derive an index to highlight these inequities for specific subgroups.

**Results:** We present an illustrative hypothetical example using simulated data for which we calculated the CVEI for non-Hispanic White, non-Hispanic Black, non-Hispanic Asian, and Hispanic patients. In the example, non-Hispanic Black and Hispanic patients had inequitable outcomes.

**Conclusion:** The index can be widely implemented to promote more equitable outcomes among racial/ethnic groups, reducing morbidity and mortality within the overall population as we pursue the collective goal of herd immunity through mass vaccination.

## Introduction

As the United States shifts its focus from COVID-19 pandemic mitigation to vaccination, health equity is at the forefront of the conversation. President Biden has placed renewed focus on COVID-19 health disparities by establishing the COVID-19 Health Equity Task Force,^1^ intended to mobilize federal resources to aid state-level health department vaccination efforts. The Centers for Disease Control and Prevention (CDC) issued vaccine allocation recommendations^2^ through the Advisory Committee on Immunization Practices, suggesting that health care workers and long-term care residents be the first to receive the vaccine. The next tier included those 75 years and older, the highest risk patient group, and gradually “high-risk” younger age groups have been included.

Despite these efforts, early evidence^3^ indicates that we are once again falling short in our efforts to prevent disparities from occurring among minoritized subgroups of the population. State governments have been assigned the ultimate authority in vaccine distribution efforts. This has resulted in inconsistencies^4^ that have impacted state-level capacities for appointments, vaccine supply, and priority setting for vaccination. These inconsistencies have also created an opportunity for further widening of disparities documented since the start of the pandemic.^5–13^

At the population level, tracking vaccine administration, disaggregated for important subpopulations, such as gender, race/ethnicity, and geography (e.g., urban vs. rural), is a crucial first step in identifying and addressing emerging disparities. However, states and the federal government are already facing challenges in this regard. On March 17, the CDC reported detailed demographic characteristics of persons vaccinated (including race/ethnicity).^14^ However, at that time, race/ethnicity was unknown or not reported for 47% of the vaccinated population. Most states^15^ use either their existing immunization information systems, CDC-supported Vaccine Administration Management System,^16^ or both systems to support their vaccine management and reporting. These systems, however, have limited ability to capture and report crucial demographic breakdowns, such as race/ethnicity where the fields are not required or simply not available. Furthermore, as of March 15, only 39 states were reporting vaccine distribution by race/ethnicity.^17^

Since the start of this pandemic, large integrated multihospital health systems^18^ have been faced with daunting tasks. Not only must they meet the rapidly evolving needs of diverse patient populations for the provision of population-based testing, treatment, and education, but now also vaccine distribution. With this central role and added responsibility, health systems have an opportunity to be proactive and mitigate barriers to vaccine access for those already disproportionately affected by the disease.

Considering these challenges, real-time electronic health record (EHR) data can and should be leveraged by key stakeholders to guide and inform policy decisions, especially at the local level where distribution decisions are made. In a recent blog in health affairs,^19^ Shete et al. state “Vaccine implementation strategies should address social and structural barriers to vaccination among populations disproportionately affected by COVID-19, target known disparities, be conducive to ‘real-time’ refinement, and mitigate any unintended negative effect on health care disparities.” EHR data are needed to inform community-based collaborations, deployment of mass vaccination and pop-up sites, and targeting of educational campaigns and outreach efforts. A data-driven approach is critical. Severe illness, hospitalizations, and deaths from COVID-19 have not been experienced equally among racial/ethnic groups in the United States. The predominant burden has fallen on communities of color. We propose to use real-time readily available EHR data to derive a vaccine equity index that accounts for disproportionate burden of illness and can be used to guide health system vaccine mitigation strategy. The index presented, building on concepts from our prior health equity index (HEI),^20^ is intended to provide insight into the impact that thoughtful vaccination strategies can have on addressing disparities. The goal is to promote more equitable outcomes among racial/ethnic groups, thus reducing overall morbidity and mortality as we pursue the collective goal of herd immunity through mass vaccination.

## Materials and Methods

### Derivation of the index

The index is focused on measuring equity, which occurs when the same patient *outcomes* are achieved regardless of sociodemographic factors (e.g., age, gender, race/ethnicity). By contrast, equality focuses on behaviors and actions, where members of all sociodemographic subgroups receive the same *treatment*. In this context, the desired outcome and a major goal of vaccination are to reduce severe illness, hospitalizations, and death. Equity is achieved when the likelihood of these adverse outcomes is comparable across all groups. The COVID-19 vaccine equity index (CVEI) identifies and quantifies disparities in outcomes for each racial/ethnic group and illustrates the potential impact of vaccine distribution on advancing equity.

The mathematical concepts used to derive the index are based in the theory of joint and conditional probabilities. We present standard notation to derive the index. We use the subscript “*s*” to denote members of a specific racial/ethnic subgroup (e.g., “*s*” might denote Hispanic) and the subscript “*t*” to denote members of the entire (or total) population at-large. Probability of an event occurring is noted by *p*(event). Therefore, the probability of a member of subgroup “*s*” being unvaccinated and the same probability for the total population at large are represented by $P\left(U_{s}\right)andP\left(U_{t}\right)$, respectively.

The index extends the notion of equality to the broader goal of equity. It is useful in our mathematical derivation to show how we start with equality and extend to measure equity. Equality is based upon taking the same actions regardless of subgroup. Here the action is vaccination. If we vaccinate all subgroups equally, the likelihood of being vaccinated (or conversely the likelihood of being unvaccinated, *U*) is the same for all subgroups.

For all subgroups, under *equality*,

$P\left(U_{s}\right)=P\left(U_{t}\right)$, and it follows that $\frac{P\left(U_{s}\right)}{P\left(U_{t}\right)}=1$, for all s.

If this ratio is >1 for a given subgroup, that group is receiving unequal and less favorable treatment, whereas a value <1 implies unequal but favorable treatment. This ratio is a building block for the index but does not measure outcomes.

To address equity, we invoke the concept of joint and conditional probabilities. A joint probability represents the probability that more than one event occurs simultaneously. For example, we are interested in identifying those individuals who are unvaccinated (*U*) and are infected with COVID-19 (*I*) and subsequently are hospitalized (*H*) with severe illness. The expression $P\left(U_{s},I_{s},H_{s}\right)$ represents the probability of a member of subgroup (s) being unvaccinated, infected, and hospitalized with severe disease. This is the term articulating the adverse outcome that the vaccine has been shown to prevent. Equity, measured by this outcome, is achieved when the likelihood of this event is the same for all racial/ethnic subgroups.

For all subgroups and total population, under *equity*,

$P\left(U_{s},I_{s},H_{s}\right)=P\left(U_{t},I_{t},H_{t}\right)$, and it follows that $P\left(U_{s},I_{s},H_{s}\right)∕P\left(U_{t},I_{t},H_{t}\right)$=1, for all *s*.

If this ratio is >1 for a given subgroup, outcomes are worse than the equitable solution; values <1 indicate outcomes that are better than an equitable solution. This ratio is the definition of the CVEI.

$$CVEI_{s}=P\left(U_{s},I_{s},H_{s}\right)/P\left(U_{s},I_{s},H_{s}\right).$$

**FIG. 1.**
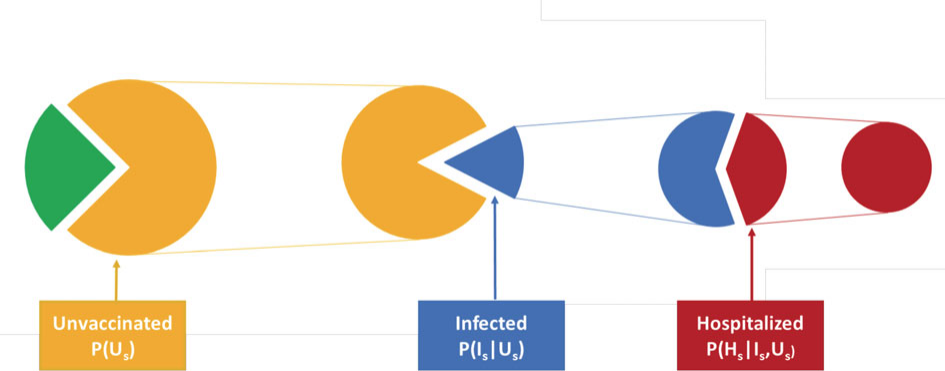
Cascade of conditional probability of hospitalization with COVID-19. Illustration of sequential pathway: (1) unvaccinated to infected among the unvaccinated; (2) infected to hospitalized among the infected and unvaccinated.

Using conditional probabilities (Fig. 1), this ratio can be deconstructed into a product of terms that are intuitive to understand, reasonably straightforward to estimate, and useful in practice. A person who is unvaccinated may become infected, then once infected may become hospitalized. Conditional probabilities measure the chance of an event happening given that certain antecedent events have already transpired. A conditional probability of interest is the chance that, given that a person in subgroup *s* is both unvaccinated (*U_s_*) and infected (*I_s_*), that individual is hospitalized (*H_s_*). This is denoted mathematically as $P\left(H_{s}|U_{s},I_{s}\right)$, where the events to the right of the vertical line are antecedent events. We are also interested in the probability that, given a person in subgroup *s* is unvaccinated, that this person goes on to be infected. This is denoted as $P\left(I_{s}|U_{s}\right)$. The chain rule of conditional probabilities^21^ permits the calculation of joint probabilities in the CVEI using only these more easily estimated conditional probabilities.

$$P\left(U_{s},I_{s},H_{s}\right)=P\left(H_{s}|U_{s},I_{s}\right)*P\left(I_{s}|U_{s}\right)*P\left(U_{s}\right).$$

Thus the CVEI can be rewritten as the product of three ratios.


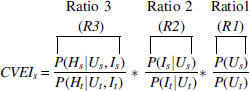


Each is a risk ratio, or ratio of probabilities comparing a specific subgroup with the total. The first risk ratio, *R*1, is a risk ratio describing our actions, the likelihood that a member of the subgroup will be unvaccinated compared with the general population, and by itself, measures equality, the extent to which we vaccinate all groups equally. The second, *R*2, compares the relative likelihood of contracting COVID-19 for subgroup *s* members who are unvaccinated compared with the general unvaccinated population. The third, *R*3, compares the relative likelihood of being hospitalized for members of the subgroup who are both unvaccinated and infected compared with members of the general population. It should be noted that this series of conditional probabilities is based on the assumption that a vaccinated person will not become infected or hospitalized with COVID-19, and it is only the unvaccinated group that is at risk for *R*2 and *R*3.

A value for the CVEI can be calculated for each distinct subgroup and be used to assess the degree to which outcomes for that subgroup achieve equity. We propose and compare two methods for estimating these risk ratios.

Method 1, using logistic regression, is more computationally complex. Using EHR data, outcomes (hospitalizations for *R*3 or infections for *R*2) among racial/ethnic subgroups can be compared using logistic regression, which allows for estimation of these probabilities while adjusting for covariates such as age, gender, clinical characteristics, socioeconomic status, and insurance status. The coefficients in the logistic regression are used to compute the relevant probabilities and risk ratios. Because this approach allows us to account for differences with respect to various characteristics among subgroups, it is a more precise way to estimate these probabilities attributed to a specific racial and ethnic subgroup.

Estimating *R*1 is straightforward, computing $P\left(U_{s}\right)$ as the ratio of the observed total number of unvaccinated people in the subgroup to the total number of individuals in the subgroup and calculating $P\left(U_{t}\right)$ similarly.

Method 2, using observed counts, provides a computationally simple way to calculate the CVEI. Although the estimates it provides are unadjusted, and, therefore, less precise, it is simple to implement, understand, and may provide insights that are directionally accurate and still useful for developing on-the-ground vaccination strategies. Estimating *R*3 numerator, $P\left(H_{s}|U_{s},I_{s}\right)$, is done using the number of subgroup *s* members with a positive COVID-19 test who are hospitalized divided by the total number of subgroups *s* who tested positive for COVID-19. The same approach is applied to the denominator to estimate $P\left(H_{t}|U_{t},I_{t}\right).$ Estimating *R*2 numerator, $P\left(I_{s}|U_{s}\right)$, is done using the number of subgroups *s* members who had a positive test result divided by the total number of subgroups *s* who were tested. The same approach is applied to the denominator, $P\left(I_{t}|U_{t}\right)$. *R*1 is estimated as above.

All computations for analytic examples were conducted using STATA version 16. This study was approved as a quality improvement initiative by the Sutter Health Institutional Review Board.

## Simulation Results

### The CVEI: an illustrative example and practical application

The CVEI and associated ratios (*R*1, *R*2, and *R*3) give insight into how to construct targeted vaccine strategies to enhance equity. The CVEI indicates whether equity has been achieved for a given subgroup and its value reflects the magnitude of the disparity. The ratios inform the specific actions we may be able to take to reduce that disparity. Elevations in *R*1 reflect equality of our vaccination efforts, whereas *R*2 and *R*3 help identify which members of the subgroup, if vaccinated, would have the greatest impact on reducing the disparity. For example, elevations in *R*2 suggest that vaccinating members of the subgroup most at risk of becoming infected would improve measures of equity. Elevations in *R*3 suggest that preferentially vaccinating subgroup members at greatest risk for severe illness and hospitalization, if infected, would have the maximum impact on improving equity.

Large health systems provide a window into a representative segment of the population within regions of the country. For illustrative purposes, using simulated data, we calculated the CVEI for non-Hispanic White, non-Hispanic Black, non-Hispanic Asian, and Hispanic patients. The indices, computed using both methods, are presented in Table 1.

**Table 1.** Illustrative Example of COVID-19 Vaccine Equity Index Calculations Using Simulated Data

Method 1—adjusted	R1	R2	R3	CVEI
Non-Hispanic White	0.95	0.78	0.89	**0.66**
Black/African American	1.17	1.19	**1.44**	**2.00**
Asian	1.02	0.84	1.24	**1.06**
Hispanic	1.10	**2.21**	1.17	**2.84**

Method 2—unadjusted	R1	R2	R3	CVEI
Non-Hispanic White	0.95	0.73	0.91	**0.63**
Black/African American	1.17	1.13	**1.42**	**1.87**
Asian	1.02	0.96	1.30	**1.27**
Hispanic	1.10	**2.42**	1.13	**3.01**

Comparison of adjusted and unadjusted methods for calculating the CVEI. Highlighted cells reflect values far in excess of equity (value much >1), and signify potential areas for intervention using targeted vaccination efforts.

Bold represents CVEI, COVID-19 vaccine equity index.

The results in Table 1 indicate that as we pursue our goal of reaching herd immunity, we would be wise to focus our outreach in the Black and Hispanic communities, groups for which vaccination must increase, if we wish to achieve equity. Furthermore, for Black communities, where the driving force is *R*3, we focus on those most likely to require hospitalization, such as elderly and those with underlying chronic conditions. For Hispanic communities, where the highest score is *R*2, we should craft mass vaccination efforts to limit the likelihood of infection.

As a practical application, if we plan to vaccinate 70% of the total population, a reasonable herd immunity goal, then 30% would potentially remain unvaccinated or *P*(*U*_t_)=0.30.

Under equity, each $CVEI_{s}=R1_{s}*R2_{s}*R3_{s}=\left[\frac{P\left(U_{s}\right)}{P\left(U_{t}\right)}\right]*R2_{s}*R3_{s}=1$.

Therefore, $P\left(U_{s}\right)=P\left(U_{t}\right)∕\left(R2_{s}*R3_{s}\right)$=0.3$∕\left(R2_{s}*R3_{s}\right)$.

In Table 2, we derive subgroup vaccine equity goals or $\left[1-P\left(U_{s}\right)\right]$ using the CVEI values in Table 1. As expected, vaccination goals for Black and Hispanic subgroups are higher than the 70% overall goal to achieve equity.

**Table 2.** Practical Application of the COVID-19 Vaccine Equity Index for Health System Vaccine Equity Goals

Method 1—adjusted Goal: 70% of total population vaccinated	Equality subgroup goals	$P\left(U_{t}\right)$	R2	R3	$P\left(U_{s}\right)$	Equity subgroup goals
Non-Hispanic White	0.70	0.30	0.78	0.89	0.43	**0.57**
Black/African American	0.70	0.30	1.19	1.44	0.18	**0.82**
Asian	0.70	0.30	0.84	1.24	0.29	**0.71**
Hispanic	0.70	0.30	2.21	1.17	0.12	**0.88**

Bold indicates equity goals.

Comparison of goals for each race/ethnicity based on a vaccination strategy of equality versus one of equity. A goal of herd immunity of 70% is assumed.

**FIG. 2.**
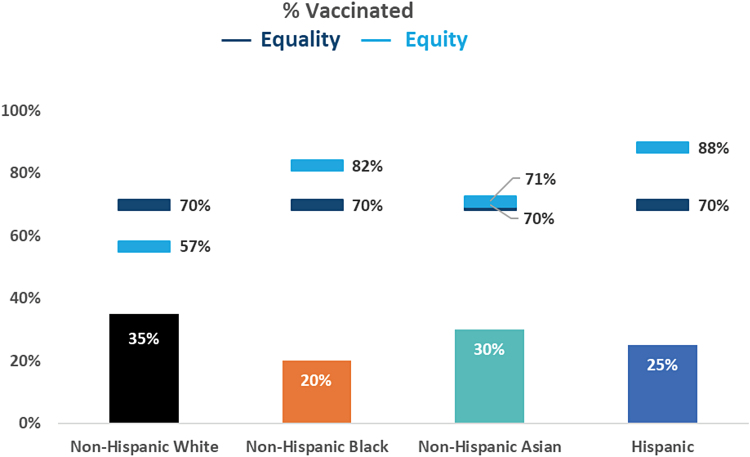
Demonstration graph of equality and equity goals by race/ethnicity. Bar graph represents percentage vaccinated for each race/ethnicity. A goal for herd immunity of 70% is assumed. Dark blue markers represent goals for each race/ethnicity based on a vaccination strategy of equality. Light blue markers represent goals for each race/ethnicity based on a vaccine strategy of equity.

In the context of the health systems, Figure 2 illustrates the two goals. First (dark blue) is subgroup equality goals. Second (light blue) is subgroup equity goals. These goals provide insight for identifying how vaccinations can be used to improve outcomes for those groups most heavily impacted by COVID-19 while striving for herd immunity.

## Discussion

COVID-19 provided a new perspective and a public emphasis on inequities that exist in our society and our health care system. Mass vaccination is a powerful tool that can lead to herd immunity among the population by decreasing the pool of those susceptible to COVID-19. All currently FDA-approved COVID-19 vaccines have shown significant reduction in likelihood of severe illness, hospitalization, and death for those vaccinated.^22–25^ The proposed CVEI is a tool that can be used to monitor and inform local strategies at the health system level for achieving herd immunity while enhancing equity by incorporating the disproportionate burden of illness into subgroup targets and goals.

COVID-19 continues to have disproportionate impact based on race/ethnicity, age, health status, occupation, and other social determinants of health.^26^ The disproportionate and untenable burden of severe illness, hospitalizations, and death borne by minoritized racial/ethnic groups has been well documented.^6,7,9,12,27^ Although calls for equitable vaccine distribution have predated the FDA's approval of available vaccines,^28–30^ little has been proposed for a data-driven approach at the local level to accomplish this goal. In 2020, the National Academies of Science, Engineering, and Medicine (NASEM) articulated a framework for equitable allocation of the COVID-19 vaccine,^30^ which advocates for a phased and tiered approach in which those most vulnerable to infection and negative health consequences are prioritized. Within each phase, all groups are given equal priority. Although this approach has been central to the CDC's guidance for national vaccine allocation,^2^ variation in policies and approaches at the state and local levels has made it challenging to implement. Some states have attempted to incorporate health equity more directly in their vaccination strategies and distribution efforts. The California Department of Public Health, for example, incorporates the California Healthy Places Index^31^ of social vulnerability to guide allocation of vaccine, prioritizing the socially and economically vulnerable. Despite this type of statewide effort to prioritize vaccination for vulnerable groups, it is the local governments and health care provider institutions that have assumed the bulk of the responsibility for vaccine administration. They have the potential to have the greatest impact on disparities, and the CVEI can assist them in developing vaccination strategies that advance equity in outcomes for COVID-19.

Given the pivotal role that large health systems are playing in implementing mass vaccination strategies, the CVEI is a pragmatic tool that leverages available EHR data to guide equitable decision making. As demonstrated in our example, the CVEI is relatively straightforward to calculate, interpret, and update. Hence, it can be employed as a dashboard metric to monitor progress and strategically allocate resources during vaccination campaigns. The real-time monitoring and use of disaggregated data to drive action^32^ at the local level are necessary to insure equity. It allows for targeting interventions to facilitate a nimble and data-driven approach to achieve quantifiable equity goals. To our knowledge, no other attempts to guide local decision making have suggested the use of an index based on EHR data.

There are some underlying assumptions and limitations in the CVEI that should be noted. First, we assumed that vaccinated patients are not likely to become infected or hospitalized. This is supported by current data regarding the performance of the vaccines available in the United States.^22–25^ Should data later emerge that vaccine effectiveness varies among subgroups, this additional complexity could be readily incorporated into a more complex version of the index. We also assume that test positivity is a reasonable proxy for infection rate. Infection rates are extremely difficult to measure and, as a result, test positivity has been routinely used as a proxy by state and local health departments and the CDC. Finally, we assume that the subset of those tested is representative of the larger population and that testing behavior among subgroups is not overly biased.

A strength of the CVEI is that the subgroup structure of this index makes it amenable to use not only in comparing racial/ethnic groups but also evaluating disparities associated with other factors such as socioeconomic status. By focusing on equity, this flexible metric extends beyond what can be achieved with equality, and minimizes severe illness and suffering as we reach herd immunity.

## Conclusion

The index is a novel innovative metric that provides information to identify and quantify disparities where they exist. Based upon concepts of equity previously deployed by the authors in the creation of the HEI,^20^ used to address disparities for ambulatory care sensitive conditions, this tool facilitates targeting of interventions to achieve high-quality equitable health care outcomes for COVID-19. It is the authors' desire that the index be further developed, validated, and widely implemented. Our hope is to provide guidance and insight for equitably vaccinating populations, and in some measure, addressing the disparities that have so clearly been demonstrated with COVID-19. The CVEI suggests that this outcome can be achieved while simultaneously reaching herd immunity.

## Abbreviations Used

ACIP: Advisory Committee on Immunization Practices
CDC: Centers for Disease Control and Prevention
COVID-19: coronavirus disease 2019
CVEI: COVID-19 vaccine equity index
EHR: electronic health record
FDA: Food & Drug Administration
HEI: health equity index
HPI: Healthy Places Index
NASEM: National Academies of Science, Engineering, and Medicine
